# Nutrient Intake and Physical Exercise Significantly Impact Physical Performance, Body Composition, Blood Lipids, Oxidative Stress, and Inflammation in Male Rats

**DOI:** 10.3390/nu10081109

**Published:** 2018-08-17

**Authors:** Richard J. Bloomer, John Henry M. Schriefer, Trint A. Gunnels, Sang-Rok Lee, Helen J. Sable, Marie van der Merwe, Randal K. Buddington, Karyl K. Buddington

**Affiliations:** 1 School of Health Studies, University of Memphis, Memphis, 106 Roane Fieldhouse, TN 38152, USA; Jschriefer44@yahoo.com (J.H.M.S.); trintagunnels@gmail.com (T.A.G.); mvndrmrw@memphis.edu (M.v.d.M.); 2 Department of Kinesiology & Dance, New Mexico State University, Las Cruces, NM 88003, USA; srlee@nmsu.edu; 3 Department of Psychology, University of Memphis, Memphis, TN 38152, USA; hjsable@memphis.edu; 4 College of Nursing, University of Tennessee Health Science Center, Memphis, TN 38152, USA; rbudding@uthsc.edu; 5 Department of Biological Sciences, University of Memphis, Memphis, TN 38152, USA; kbudding@memphis.edu

**Keywords:** dietary restriction, macronutrients, physical exercise, free radicals, cytokines

## Abstract

Background: Humans consuming a purified vegan diet known as the "Daniel Fast" realize favorable changes in blood lipids, oxidative stress, and inflammatory biomarkers, with subjective reports of improved physical capacity. Objective: We sought to determine if this purified vegan diet was synergistic with exercise in male rats. Methods: Long–Evans rats (*n* = 56) were assigned to be exercise trained (+E) by running on a treadmill three days per week at a moderate intensity or to act as sedentary controls with normal activity. After the baseline physical performance was evaluated by recording run time to exhaustion, half of the animals in each group were fed ad libitum for three months a purified diet formulated to mimic the Daniel Fast (DF) or a Western Diet (WD). Physical performance was evaluated again at the end of month 3, and body composition was assessed using dual-energy x-ray absorptiometry. Blood was collected for measurements of lipids, oxidative stress, and inflammatory biomarkers. Results: Physical performance at the end of month 3 was higher compared to baseline for both exercise groups (*p* < 0.05), with a greater percent increase in the DF + E group (99%) than in the WD + E group (51%). Body fat was lower in DF than in WD groups at the end of month 3 (*p* < 0.05). Blood triglycerides, cholesterol, malondialdehyde, and advanced oxidation protein products were significantly lower in the DF groups than in the WD groups (*p* < 0.05). No significant differences were noted in cytokines levels between the groups (*p* > 0.05), although IL-1β and IL-10 were elevated three-fold and two-fold in the rats fed the WD compared to the DF rats, respectively. Conclusions: Compared to a WD, a purified diet that mimics the vegan Daniel Fast provides significant anthropometric and metabolic benefits to rats, while possibly acting synergistically with exercise training to improve physical performance. These findings highlight the importance of macronutrient composition and quality in the presence of ad libitum food intake.

## 1. Introduction

Both dietary restriction/modification and caloric restriction have been studied extensively for their ability to favorably alter body composition in humans and animals [1]. One form of dietary restriction is veganism, a plan that eliminates all animal products and improves health outcomes [2]. Unlike caloric restriction, which typically calls for a 10–30% reduction in daily dietary energy needs [1], veganism allows for ad libitum food intake. 

The Daniel Fast (DF), a biblically inspired partial fast, is similar to veganism but much more stringent. Specifically, the DF is a form of dietary restriction that allows for ad libitum food intake but places firm restrictions on the types of food that are allowed [1,3,4,5], with choices primarily limited to fruits, vegetables, whole grains, legumes, nuts, seeds, and plant-based oils. No alcohol, sweeteners, or refined foods are allowed, resulting in carbohydrate sources that are complex with low glycemic indices. By default, the DF has an abundance of dietary fiber and plant-derived fatty acids and relatively high concentrations of antioxidants. 

Our previous human studies involving the DF revealed health-specific benefits including, but not limited to, reductions in body mass and body fat, systolic and diastolic blood pressure, total and LDL-cholesterol, blood oxidative stress biomarkers, and C-reactive protein [3,4,6]. The DF has also been shown to increase antioxidant capacity as well as the ratio of blood nitrate/nitrite (a biomarker of nitric oxide), which may have implications for improving physical performance with enhanced hemodynamic responses [7,8]. While we received multiple anecdotal reports of improved vitality, vigor, and mood from research participants in these studies [3,4,6], objective physical performance measures were not investigated in a controlled study. It is possible that chronic consumption of the DF with and without regular exercise—which is well known to aid in body mass/fat control and to improve functional capacity [9,10,11]—may yield favorable changes in body mass/fat and physical performance. 

The present study sought to determine the independent and combined influences of dietary composition (under conditions of ad libitum intake) and moderate exercise training on functional capacity, body composition, blood lipids, oxidative stress, and inflammation in male rats. The decision to use an animal model was to ensure the control of key variables known to impact outcome measures (e.g., sleep, stress, type and volume of food, volume and intensity of exercise). We hypothesized that exercise training would improve physical performance as well as other outcome measures and that the benefits of exercise would be of greater magnitude in animals fed the DF. 

## 2. Materials and Methods 

### 2.1. Overview of the Experimental Design 

Male Long–Evans rats (*n* = 56), 3–4 weeks of age, were purchased from Harlan Laboratories, Inc. (Indianapolis, IN, USA). Upon arrival, all rats were individually housed in standard shoebox caging in a climate-controlled room (21 °C), employing a standard 12:12 h light–dark cycle (lights on at 8:00 h). During a two-week acclimation period, they were transitioned from consuming a standard rat chow (Harlan 1018) to their assigned diets by gradually replacing the standard chow diet with an increasing proportion of the experimental diets. During this two-week period, the rats were also familiarized with the treadmill on three separate days (i.e., walking on the treadmill for 5 min at 15–20 m∙min^−1^), and the 12:12 h light–dark cycle was progressively shifted to lights on at 300 h and off at 1500 h. The light–dark cycle was shifted to allow exercise training and testing to occur during the latter part of the light phase. All housing and experimental procedures were approved by The University of Memphis Institutional Animal Care and Use Committee (approval #0734) and were in accordance with the 8th edition of the *Guide for the Care and Use of Laboratory Animals.* Throughout the study, the rats had ad libitum access to food and water.

### 2.2. Functional Capacity Assessment

The pre-Intervention (Baseline) total treadmill running time was determined for all animals after the two-week acclimation period following established procedures [12,13,14]. The animals began the test by running on a motorized treadmill (Exer-6M Treadmill, Columbus Instruments, Columbus, OH, USA) at a speed of 20 m∙min^−1^ without incline for 15 min. The speed was increased by 5 m∙min^−1^ every 15 min to a maximum speed of 35 m∙min^−1^. A mild electrical shock (frequency current at 3.0 hz at 1.60 mA with a voltage of a 115) was provided when the animals could not maintain the set pace. Fatigue was considered to occur when a rat started to lower its hindquarters and raise its snout, resulting in a significantly altered gait, to the point of not being able to remain on the treadmill. When this degree of fatigue was noted, and the animal had difficulty remaining on the treadmill belt (regardless of the delivery of the electrical shock), the animal was taken off the treadmill, and the run time was recorded to the nearest second. Again, testing was performed during the latter part of the light phase, when the rats are the most active [15]. All rats repeated the same treadmill test at the end of the intervention period.

### 2.3. Anthropometric Assessments

Body mass was measured daily. At the end of the three-month intervention, the body composition of all animals, anesthetized with isoflurane, was evaluated by using dual-energy x-ray absorptiometry (DXA) (Discovery QDR series, Hologic Inc., Bedford, MA, USA). The reliability of the DXA exam was evaluated using three rats not participating in the study that were scanned a total of seven times each. The measured variance of the percent body fat for these animals was 0.34, 0.60, and 0.89. Despite the low variance, all experimental animals were scanned twice. If the first two scans provided percent body fat values that varied by more than 1.5%, a third scan was performed, the two scans that were closest were averaged, and the mean value of these two scans was included in the data analysis. These data were used to calculate lean body mass, fat mass, and percent body fat. 

### 2.4. Dietary and Exercise Interventions

The rats were randomly assigned to one of four intervention groups: Western Diet with exercise (WD + E; *n* = 14); Western Diet without exercise (WD; *n* = 14); Daniel Fast with exercise (DF + E; *n* = 14); Daniel Fast without exercise (DF; *n* = 14). The diets were purchased from Research Diets, Inc. (New Brunswick, NJ) and provided in pellet form. The WD was formulated to mimic a typical human WD, containing 17% protein, 43% carbohydrates, and 40% fat—A large portion of which was saturated (milk fat) (product: D12079B). The DF included 15% protein, 60% carbohydrates, and 25% fat (product: D13092801). The macronutrient sources and quantities of the DF were based on the dietary intakes of human subjects in our prior DF studies [1,3,4]. The DF diet fed to the rats therefore mimics what our human subjects consumed in terms of macronutrient sources and composition, fiber, and micronutrients (i.e., antioxidants). The specific nutrient compositions of the WD and DF are provided in Table 1.

The rats consumed their assigned diet (WD or DF) for three months, beginning after the two-week acclimation period. Food was provided ad libitum and was not measured each day. We simply monitored the animals’ body mass over time. Our omission of daily food recording may be considered a limitation of this work. Equal numbers of rats in each diet group were randomly assigned either exercise (+E) or no exercise. The animals in the no-exercise groups were placed on the treadmill three days per week for a period of 5 min, while it was turned off. The animals in the exercise groups performed moderate-intensity endurance exercise on a motorized treadmill three days per week (i.e., Monday, Wednesday, Friday) for the three-month intervention period. The speed and duration was progressively increased. Specifically, the animals began training at 20 m∙min^−1^ for 15 min∙day^−1^ (week 1), progressed to 25 m∙min^−1^ for 30 min∙day^−1^ (week 2), and then to 25 m∙min^−1^ for 35 min∙day^−1^ (weeks 3–12). This progressive increase in intensity and duration of exercise is typical for rodent training studies [16].

### 2.5. Blood Collection and Analysis

At the end of the three-month intervention, one half of the animals in each group were euthanized, with the remaining animals being retained for a separate long-term study. For blood collection, the rats were euthanized via CO_2_ inhalation, the abdominal cavity was opened, and the blood was collected from the inferior vena cava, using a syringe with a 22-gauge needle, and placed in vacutainer tubes containing EDTA. Plasma was separated, and multiple aliquots were stored at −70 °C for the analysis of plasma lipids and biomarkers of oxidative stress and inflammation. Plasma triglycerides (TAG) and total cholesterol were analyzed following standard enzymatic procedures, as described by the reagents’ manufacturer (Thermo Electron Clinical Chemistry; product #: TR22421 and TR13421 for TAG and cholesterol, respectively). Malondialdehyde (MDA) was analyzed following the procedures of Jentzsch and colleagues [17], using reagents purchased from Northwest Life Science Specialties (Vancouver, WA; product #: NWK-MDA01). Advanced Oxidation Protein Products (AOPP) were measured using the methods described by the reagent’s manufacturer (Cell Biolabs, Inc., San Diego, CA, USA; product #: STA-318). A customized Milliplex^®^ Map Kit was purchased from the EMD Millipore Corporation (Billerica, MA, USA) for the measurement of IL-1α, IL-4, IL-1β, IL-6, IL-13, IL-10, IFNγ, and TNF-α. The cytokine concentrations were determined using the MAGPIX^®^ platform with xPONENT software (MilliporeSigma, Burlington, MA, USA). All samples were analyzed in duplicate. Since blood was only obtained at the time of death, no baseline values are available for the biochemical measures.

### 2.6. Statistical Analysis

The treadmill run time was analyzed using a 4 (group) × 2 (time) analysis of variance (ANOVA). The data obtained from the DXA scan (fat mass, lean mass, percent body fat) and all biochemical measures were analyzed using a one-way ANOVA. Tukey post-hoc tests were used to identify significant group differences. All analyses were performed using JMP statistical software (version 4.0.3; SAS Institute; Cary, NC, USA). Statistical significance was set at *p* ≤ 0.05. All data are expressed as the mean ± SEM.

## 3. Results

One animal in the WD + E group died during week two of the intervention, approximately 30 min following the exercise training session. The necropsy revealed the abdomen was filled with blood, with a suspected aneurism. All remaining animals completed the three-month intervention.

### 3.1. Physical Performance Data

After three months, the treadmill run time to exhaustion (Table 2) displayed a significant group effect (WD + E & DF + E) > (WD & DF) (*p* < 0.0001) and a significant time effect (month 3 > baseline) (*p* = 0.0005), as well as a group x time interaction effect (*p* < 0.0001). The run time increased in both exercise groups, as demonstrated by a change in the percent improvement from baseline to month 3 in the WD + E (+51%) and the DF + E (+99%) groups. The increase for the DF + E group exceeded that for the WD + E group (*p* = 0.02).

### 3.2. Anthropometric Data

All the rats gained weight during the three-month study (*p* < 0.0001; Table 3). The gain in body weight displayed a group effect (*p* < 0.0001), with the 205% increase in the WD group exceeding (*p* < 0.05) those in the WD + E (177%), DF + E (148%), and DF (168%) groups. There was no difference between DF and WD + E rats for body weight gain (*p* > 0.05). A group effect was noted for mean fat mass (*p* < 0.0001), with DF rats having a lower mean fat mass than the WD groups; also, mean fat mass in WD + E rats was lower than in WD rats (*p* < 0.05). A group effect was noted for body fat percentage (*p* < 0.0001), with both DF groups displaying lower values than the WD groups; also, body fat percentage in WD + E rats was lower than in WD rats (*p* < 0.05). There was no significant difference in lean mass between the groups (*p* = 0.14). 

### 3.3. Biochemical Data

**Figure 1:**
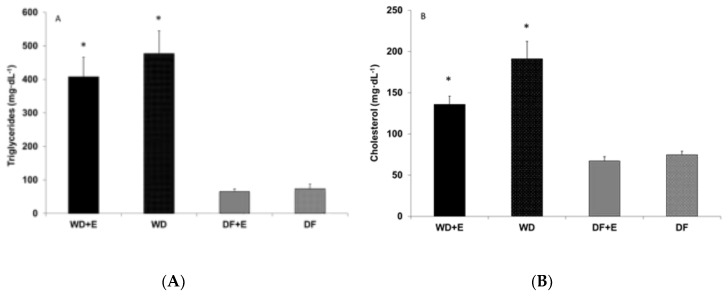
(**A**) Blood triglycerides (TAG) and (**B**) total cholesterol of male rats assigned to two different diets with and without exercise. Values are mean ± SEM. *A group effect was noted for TAG (*p* < 0.0001) and cholesterol (*p* < 0.0001). For TAG and cholesterol, the Western Diet (WD) group was different from all the other groups, and the Western Diet + Exercise (WD + E) group was different from all the other groups (*p* < 0.05).

**Figure 2:**
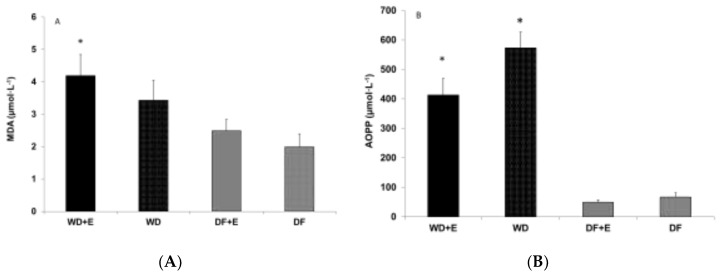
(**A**) Blood malondialdehyde (MDA) and (**B**) advanced oxidation protein products (AOPP) of male rats assigned to two different diets with and without exercise. Values are mean ± SEM. * A group effect was noted for MDA (*p* = 0.03) and AOPP (*p* < 0.0001). For AOPP, the Western Diet (WD) group resulted different from all the other groups; also the Western Diet + Exercise (WD + E) group resulted different than all the other groups (*p* < 0.05). For MDA, the Western Diet + Exercise (WD + E) group resulted different from the DF group (*p* < 0.05).

Since only one half of the animals were euthanized at the end of month 3, only samples from these animals were available for biochemical analysis (*n* = 7 per group). A group effect was noted for TAG (*p* < 0.0001; Figure 1), cholesterol (*p* < 0.0001; Figure 1), MDA (*p* = 0.03; Figure 2), and AOPP (*p* < 0.0001; Figure 2). The values for TAG, cholesterol, and AOPP in the WD group were different from those in all other groups (Figure 1 and Figure 2B); also, the values in the WD + E group were different from those in all the other groups (*p* < 0.05). For MDA, the WD group displayed a higher value than the DF + E group (*p* < 0.05).

The cytokine results are presented in Table 4. The concentrations of four of the examined cytokines were below the limit of detection and were not included. Due to variation between animals, the differences in IL-1β and IL-10 concentrations in the WD group compared with the DF rats did not reach statistical significance (*p* > 0.05). The concentrations of the remaining cytokines were similar in both groups.

## 4. Discussion 

To our knowledge, this is the first study to investigate the impact of a purified diet that mimics the Daniel Fast, with and without exercise training, on physical performance, body composition, and blood-derived measures of health in rats. Our data clearly indicate that a purified vegan diet improves body composition, blood lipids, and measures of oxidative stress compared to a WD. In addition, exercise training for three months enhances physical performance but has little impact on blood lipids or oxidative stress when consuming the DF, with some noted improvement in blood lipids and AOPP when consuming the WD.

### 4.1. Physical Performance Findings

Several studies have previously reported that exercise training increases endurance physical performance of rats [16,18,19,20]. This led to the a priori expectation that the exercise training groups would exhibit better physical performance compared to the sedentary groups, which was confirmed. Our hypothesis that rats fed the DF would display a greater adaptive response to exercise than rats fed the WD was demonstrated by the respective 99% versus 51% increases in run times for the DF + E and WD + E groups. These findings are also in agreement with other studies indicating that a Western-style diet can blunt exercise-induced performance gain [21,22,23,24]. It is possible that a much higher volume or intensity of exercise may have yielded more robust findings for physical performance and associated variables, as the present study employed only a moderate intensity and volume of exercise. 

Our prediction was that the WD with and without exercise would impair endurance performance because of its relatively high fat content, which over time may lead to increased fat mass, which has been linked with higher oxygen consumption and perceived exertion during exercise [25,26]. Moreover, high-fat diets have been implicated in impaired glucose metabolism by skeletal muscle [27], which is important for muscle oxidation during exercise [28]. Diets with high levels of saturated and monounsaturated fat and low levels of polyunsaturated fatty acids, such as the WD used in the present study, yield poor exercise performance in both animals and humans [22,29]. The DF diet provided low levels of saturated and monounsaturated fatty acids and high levels of polyunsaturated fatty acids and was associated with improved exercise performance. 

In addition to the amount and form of dietary fat, the amount and type of carbohydrate may have contributed to our findings. High-carbohydrate diets increase time to exhaustion [30,31] by delaying the complete oxidation of muscle glycogen [30,32]. The complex low-glycemic carbohydrates in the DF have been reported to enhance physical performance [33,34]. Overall, it is conceivable that the differences in the amount and form of dietary fat and carbohydrate may have contributed to the divergence in physical performance for the WD and DF rats. 

### 4.2. Anthropometric Findings

Despite ad libitum feeding in all groups, body mass was greater in the WD group than in all others. We anticipated that the exercise groups would have a lower overall body mass due to the increased caloric expenditure from the thrice-weekly exercise bouts [35]. However, DF animals, despite being allowed to freely feed throughout the three-month intervention period, showed only a slightly reduced body mass compared to animals in the WD groups; in contrast, they showed a remarkably lower body fat. Despite the dramatic difference in body fat between the WD and DF groups, only small differences were noted in lean body mass. These findings suggest that animal protein is not necessary for the development of lean body mass—an often expressed concern raised by many nutritionists. It should be noted that, despite the plant-based protein sources, all DF animals grew as expected over the course of the three-month period and, importantly, without accumulating the excessive amounts of fat measured in the WD groups. 

### 4.3. Biochemical Findings

In agreement with our findings obtained from human subjects [3,4,6], blood lipids and oxidative stress biomarkers were lower in the DF groups than in the WD groups. In terms of plasma lipids, the six-fold and two- to three-fold higher TAG and cholesterol values in the WD rats compared to the DF rats are attributed to differences in the types and amounts of dietary carbohydrate (including fiber) and fat and are in line with the 20% reduction in total and LDL-cholesterol measured in human subjects after just 21 days of adherence to the Daniel Fast [3,6]. 

The differences between the WD and DF groups for both MDA and AOPP are indicative of differences in lipid peroxidation and protein oxidation. The approximately eight-fold decrease in AOPP in rats fed the DF exceeds the decrease in oxidative stress observed in human subjects following the Daniel Fast for 21 days [4]. This suggests that maintaining a DF regimen beyond 21 days can continue the decline in oxidative burden, but this hypothesis needs to be confirmed, including other measures of oxidative stress. While exercise training improved physical performance in both exercise groups, chronic ingestion of the WD appears to blunt the exercise-driven improvement. This observed response may be at least partially attributable to higher levels of oxidative stress. Increased oxidative stress alters protein function by damaging both contractile (primarily myosin, due to thiol group oxidation [36]) and enzymatic proteins, compromising the excitation–contraction coupling [37] and potentially slowing reaction rates, respectively. Moreover, the potential oxidation of mitochondrial enzymes required for energy production (e.g., succinate dehydrogenase, cytochrome oxidase [38]) could negatively impact endurance performance. 

Although systemic inflammation is reduced in human subjects following the Daniel Fast for 21 days [3,6,39], this was not evident in the rats in the present study. The human studies suggest that, with larger samples sizes, the approximately three-fold and two-fold higher concentrations of IL-1β and IL-10, respectively, in the WD rats may have reached significance. This is important, as IL-1β is secreted by all nucleated cells, including macrophages, monocytes, B cells, fibroblasts, chondrocytes, and keratinocytes [40], and has been repeatedly labeled as one of the most significant factors in regulating both the local and the systemic onset of acute and chronic inflammation [41,42]. The cytokine patterns found in the different groups also confirms previous research indicating that physical inactivity coupled with a WD leads to higher inflammatory levels [43,44]. The higher IL-10 in the WD groups may have been due to a compensatory response to increased pro-inflammatory cytokines (e.g., IL-1β). More work is needed to investigate this possibility. 

## 5. Conclusions

The present work is unique in investigating the effect of a purified diet, with and without exercise, on measures of functional capacity, body composition, and biochemical outcomes in male rats. The findings extend our prior work in human subjects partaking in the Daniel Fast dietary plan and emphasize that macronutrient composition, and not simply calorie intake, has an influence on the degree of adiposity, independent of exercise. Notably, compared to the glycemic- and saturated fatty acid-rich WD, the macronutrient mix (low levels of glycemic carbohydrate, high levels of polyunsaturated fat) of the DF, significantly improves body composition, plasma lipids, markers of oxidative stress, and physical performance as measured by treadmill run time to exhaustion. Future investigations using animal models and human subjects are needed to define the specific mechanisms responsible for the improved indicators of health and physical performance in response to the Daniel Fast dietary plan, with and without exercise. 

## Figures and Tables

**Table 1:** Dietary composition of the Western Diet and Daniel Fast.

Nutrient	Western Diet		Daniel Fast	
	gm%	kcal%	g%	kcal%
Protein	20	17	15	15
Carbohydrate	50	43	58	59
Fat	21	40	11	25
Fiber	5	0	13	1
Total		100		100
kcal/g	4.7		3.9	
Casein	195	780	0	0
Soy Protein	0	0	170	680
DL-Methionine	3	12	3	12
Corn Starch	50	200	0	0
Corn Starch-Hi Maize 260 (70% Amylose and 30% Amylopectin)	0	0	533.5	2134
Maltodextrin 10	100	400	150	600
Sucrose	341	1364	0	0
Cellulose, BW200	50	0	100	0
Inulin	0	0	50	50
Milk Fat, Anhydrous	200	1800	0	0
Corn Oil	10	90	0	0
Flaxseed Oil	0	0	130	1170
Ethoxyquin	0.04	0	0.04	0
Mineral Mix S1001	35	0	35	0
Calcium Carbonate	4	0	4	0
Vitamin Mix V1001	10	40	10	40
Choline Carbonate	2	0	2	0
Ascorbic Acid Phosphate, 33% active	0	0	0.41	0
Cholesterol	1.5	0	0	0
Total	1001.54	4686	1187.95	4686
Saturated g/kg	122.6		7.8	
Monunsaturated g/kg	60.2		19.7	
Polyunsaturated g/kg	13.5		77.7	
Cholesterol mg/kg	2048		0	
Saturated %Fat	62.4		7.4	
Monunsaturated %Fat	30.7		18.7	
Polyunsaturated %Fat	6.9		73.9	
Ascorbic Acid mg/kg	0		114	

**Table 2:** Treadmill run time (min) to exhaustion of male rats assigned to two different diets with and without exercise.

	Western Diet + Exercise (WD+E)	Western Diet (WD)	Daniel Fast + Exercise (DF+E)	Daniel Fast (DF)
Pre-Intervention (Baseline)	35.5 ± 3.5	28.7 ± 3.3	29.3 ± 2.8	33.6 ± 4.1
Post-Intervention **	48.3 ± 1.9 *^,†^	24.4 ± 1.5	52.9 ± 1.9 *^,†^	28.8 ± 1.1

Values are mean ± SEM. The data show: a group effect for treadmill run time to exhaustion (*p* < 0.0001); * WD + E & DF + E > W & DF; a time effect for treadmill run time to exhaustion (*p* = 0.0005); ** Month 3 > Baseline, a group-by-time interaction effect for treadmill run time to exhaustion (*p* < 0.0001). ^†^ Month 3 > Baseline for WD + E and DF + E.

**Table 3:** Anthropometric data of male rats assigned to two different diets with and without exercise.

	Western Diet + Exercise	Western Diet	Daniel Fast + Exercise	Daniel Fast
Body Mass (g) *Pre-Intervention*	186.5 ± 3.3	187.0 ± 4.5	192.6 ± 2.7	185 ± 4.8
Body Mass (g) *Post-Intervention*	516.8 ± 10.7	571.1 ± 14.7	478.7 ± 11.3	496.8 ± 13.5
Fat Mass (g) *Post-Intervention*	161.6 ± 8.0	195.5 ± 8.4	100.73 ± 7.4	124.45 ± 9.8
Lean Mass (g) *Post-Intervention*	366.0 ± 9.2	386.8 ± 6.7	391.4 ± 8.8	376.5 ± 7.8
% Fat *Post-Intervention*	30.6 ± 1.3	33.5 ± 1.0	20.3 ± 1.3	24.6 ± 1.4

Values are mean ± SEM. A group effect was noted for body mass (*p* < 0.0001). A time effect was noted for body mass (*p* < 0.0001). A group-by-time interaction effect was noted for body mass (*p* < 0.0001). A group effect was noted for fat mass (*p* < 0.0001). A group effect was noted for % fat (*p* < 0.0001). No other statistically significant effects were noted (*p* > 0.05).

**Table 4:** Cytokine concentrations in male rats assigned to two different diets with and without exercise.

Variable	Western Diet + Exercise	Western Diet	Daniel Fast + Exercise	Daniel Fast
IL-4	37 ± 9.6	25.9 ± 7.8	36.3 ± 9.6	27.1 ± 9.6
IL-1β	174.5 ± 59.5	120.9 ± 55.1	31.4 ± 59.5	72.3 ± 65.2
IL-10	126.5 ± 36.7	95.5 ± 34	40.4 ± 34	63.4 ± 34
TNF-α	14.3 ± 5.1	13.8 ± 4.1	14.6 ± 3.6	12.6 ± 4.1

Values are mean ± SEM (pg∙mL^−1^). No statistically significant differences were noted (*p* > 0.05).

## Abbreviations

**AOPP**	Advanced Oxidation Protein Products
**DF**	Daniel Fast
**DXA**	Dual-Energy X-ray Absorptiometry
**+E**	Exercise-Trained
**MDA**	Malondialdehyde
**TAG**	Triglycerides
**WD**	Western Diet
